# Analysis of risk factors of Mycoplasma pneumoniae infection in children

**DOI:** 10.1097/MD.0000000000048381

**Published:** 2026-05-01

**Authors:** Kun Zhang, Xiaodan Zhai, Ying Zhang, Xiaolin Xu, Xuejing Wang

**Affiliations:** a Clinical laboratory, Civil Aviation General Hospital, Chaoyang, Beijing, China.

**Keywords:** children, MP infection, predictive factors, the neutrophil-to-lymphocyte ratio (NLR)

## Abstract

To explore the risk factors and predictive value of Mycoplasma pneumoniae (MP) infection in children. A total of 2042 children with suspected Mycoplasma pneumoniae infection who were treated for the first time at Civil Aviation General Hospital from October 2023 to December 2023 were selected as the study subjects. Among them, 1637 cases were confirmed as Mycoplasma pneumonia-infected and were included in the pneumonia group, while the remaining 405 cases were non-Mycoplasma pneumonia-infected and were included in the non-Mycoplasma pneumonia group. The clinical data of the 2 groups of children (including gender, age, initial symptoms, laboratory indicators, etc) and the risk factors of MP infection in children were compared, and the receiver operating characteristic curve was analyzed. This study showed that the percentage of neutrophils in the non-MP infection group was significantly lower than that in the MP infection group, and the difference was statistically significant (*P* < .001). When comparing the percentages of lymphocyte percentage (LY) and hemoglobin in the 2 groups of children, the Mycoplasma pneumonia-infected group was lower than the non-Mycoplasma pneumonia-infected group, and the differences were both statistically significant (*P* < .05). Logistic regression analysis revealed that white blood cell and neutrophil-to-lymphocyte ratio (NLR) might be valuable markers for predicting MP infection. The Spearman correlation indicated that LY was collinear with the occurrence of MP infection, and Least Absolute Shrinkage and Selection Operator (LASSO) regression analysis demonstrated that both LY and NLR might be valuable markers for predicting MP infection (*P* < .05). Receiver operating characteristic curve analysis revealed that the area under the curve of the NLR for diagnosing MP infection was 0.624, with a cutoff value of 1.36 (sensitivity of 0.798 and specificity of 0.558). In the diagnosis of MP infection, the consistency between the RNA method and the immunogold colloidal method was poor (Kappa = 0.108, *P* < .05). The consistency between the RNA method and the immunogold colloidal method in the diagnosis of MP infection is poor. Both the white blood cell and NLR are valuable markers for MP infection.

## 1. Introduction

Lung inflammation caused by Mycoplasma pneumoniae (MP) infection is called Mycoplasma pneumoniae pneumonia (MPP). It is the most common type of community-acquired pneumonia among children aged 5 and above in China.^[1]^ It is mainly transmitted through droplets and has a higher incidence in autumn and winter.^[2–4]^ Its clinical characteristics include a long course of disease, rapid progression of pulmonary lesions, poor treatment effect, and various extrapulmonary complications. If not diagnosed and treated early, it is likely to cause respiratory tract inflammation, further damage lung function, and in severe cases, can trigger asthma, leading to disability or death.^[5]^

Therefore, early identification and early intervention of MPP are particularly necessary. Currently, the etiological and serological examinations for MPP include MP culture, MP nucleic acid detection, and MP antibody determination. MP culture is the “gold standard” for diagnosing MP infection. However, due to the special conditions required for MP culture and its slow growth, it is difficult to use it in clinical diagnosis. The detection of MP nucleic acids, which encompasses MP-DNA and MP-RNA, boasts high sensitivity and specificity. This makes it an ideal method for the early diagnosis of MPP. When it comes to MP antibody testing, the MP-IgM antibody typically emerges 4–5 days post-infection. It can serve as a diagnostic marker for the initial phase of infection. Among them, immunogold colloidal detection can qualitatively detect MP-IgM antibody. A positive result indicates MP infection, while a negative result does not completely rule out MP infection. It is suitable for rapid screening of children in outpatient and emergency departments, but false-positive results may also occur, and comprehensive analysis should be carried out in combination with clinical and imaging features.^[1]^

A few studies at home and abroad have revealed the consistency of serological detection methods for MP. Currently, the risk factors for the early diagnosis of MP infection in children remain unclear. This study aims to compare the diagnostic consistency of the nucleic acid method and immunogold colloid method for MPP, and further clarify the related risk factors of MP infection.

## 2. Materials and methods

### 2.1. Participants

**Figure 1.**
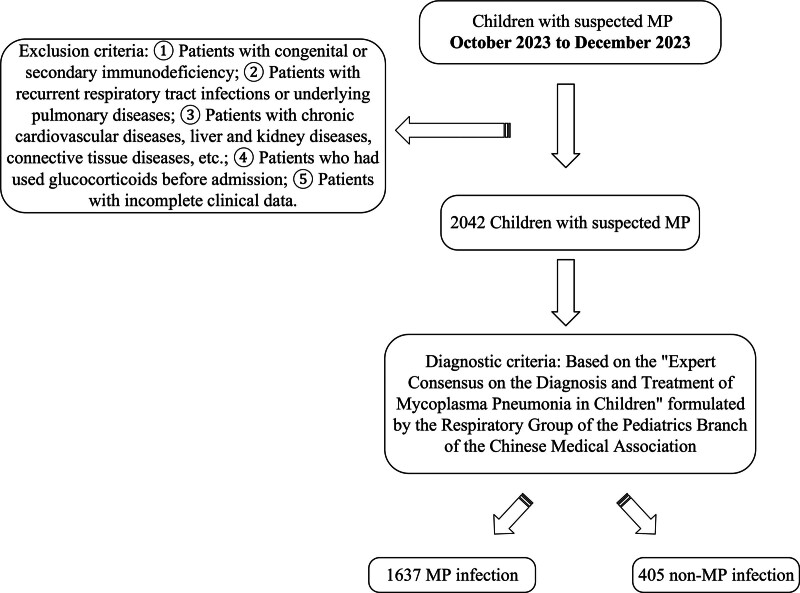
Flow diagram of patient recruitment and exclusion.

This research was approved by the Ethics Committee of Civil Aviation General Hospital. A total of 2042 children with suspected MP infection who were treated for the first time at Civil Aviation General Hospital from October 2023 to December 2023 were selected as the study subjects. Among them, 1637 cases were confirmed as MP infection and were included in the pneumonia group, while the remaining 405 cases were non-MP infection and were included in the non-pneumonia group. Diagnostic criteria, based on the “Expert Consensus on the Diagnosis and Treatment of Mycoplasma Pneumonia in Children,” were formulated by the Respiratory Group of the Pediatrics Branch of the Chinese Medical Association.^[6]^ Exclusion criteria: Patients with congenital or secondary immunodeficiency; Patients with recurrent respiratory tract infections or underlying pulmonary diseases; Patients with chronic cardiovascular diseases, liver and kidney diseases, connective tissue diseases, etc; Patients who had used glucocorticoids before admission; and Patients with incomplete clinical data. The patient inclusion and exclusion process is shown in Figure 1.

### 2.2. Data collection

Prospectively collect the general clinical information of patients, including age, gender, clinical symptoms, and signs. The laboratory test results at the time of visit, including white blood cell (WBC) counts, hemoglobin (HGB), platelets, lymphocyte percentage (LY), neutrophil percentage, monocyte percentage, neutrophil-to-lymphocyte ratio (NLR), C-reactive protein (BC-7500[NR]CS; Mindray, Shenzhen, China), were recorded.

### 2.3. Statistical analysis

Data were statistically analyzed using R version 4.4.1 and Graphpad Prism 8 software. For data conforming to a normal distribution, continuous variables were described as mean ± standard deviation (X ± S). A *t* test was used for comparisons between 2 groups. Categorical variables were described by the number of cases (n) and percentage (%), and a Chi-square test was applied. *P* < .05 was considered statistically significant.

## 3. Results

### 3.1. Demographic and baseline clinical characteristics of patients with MP infection and non-MP infection

**Figure 2.**
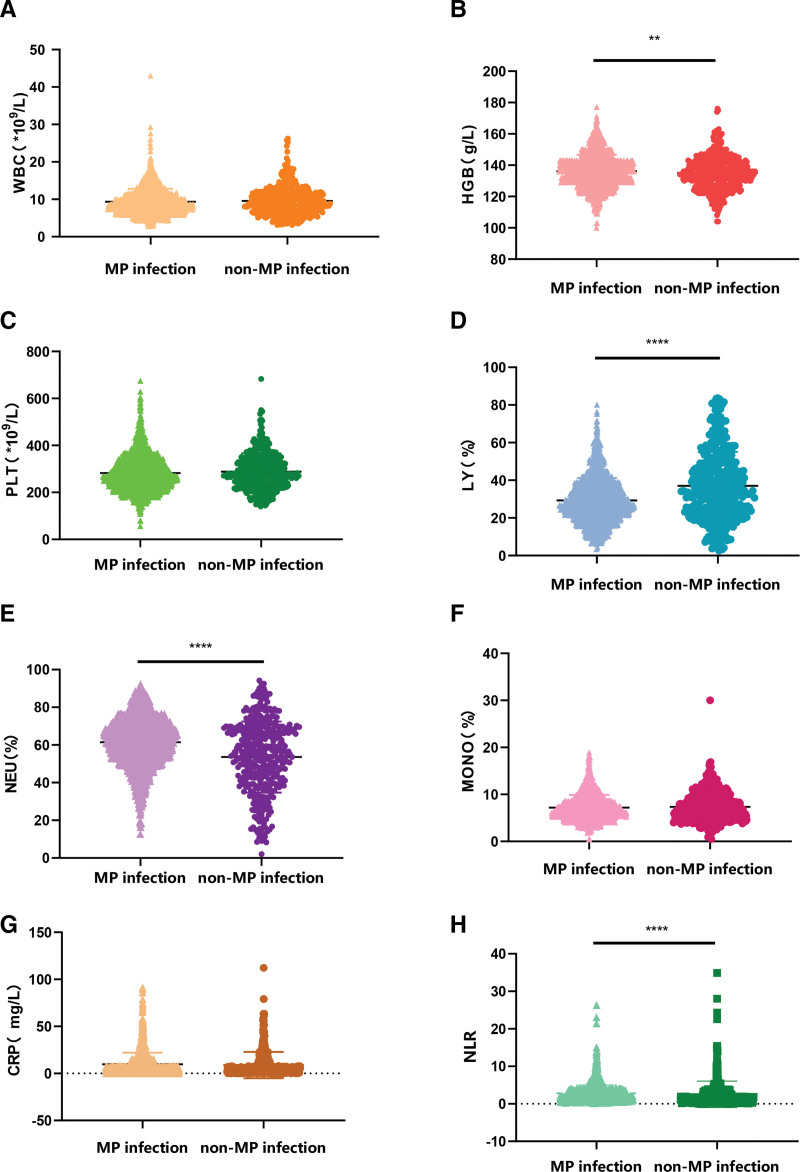
Comparison of laboratory tests between mycoplasma pneumoniae group and non-mycoplasma pneumoniae group. WBC: white blood cell counts, HGB = hemoglobin, PLT = platelets, LY = lymphocyte percentage, NEU = neutrophil percentage, MONO = monocyte percentage, NLR = neutrophil-to-lymphocyte ratio, CRP = C-reactive protein. *P < .05; **P < .01; ***P < .001; ****P < .0001.

The basic demographic information and baseline clinical characteristics of 2042 patients are shown in Table 1. The percentage of neutrophils in the non-MP infection group was lower than that in the MP infection group, and the difference was statistically significant (*P* < .001). When comparing the percentages of LY and hemoglobin between the 2 groups of children, the percentages in the MP infection group were lower than those in the non-MP infection group, and the differences were statistically significant (*P* < .05). (Fig. 2) However, although hemoglobin showed a statistically significant difference between the 2 groups (136 vs 139 g/L), the absolute mean difference was small. This suggests that the clinical relevance of HGB in MP infection may be limited, and the result should be interpreted with caution.

**Table 1:** Demographic and baseline clinical characteristics of patients with MP infection and non-MP infection.

	MP infection (1637)	non-MP infection (405)	*X*^2^/*t*	*P*
Gender			0.03	.865
Male	887 (54%)	43 (57%)		
Female	750 (46%)	32 (43%)		
Age			0.67	.505
≤3	192 (12%)	10 (13%)		
4–6	377 (23%)	14 (19%)		
7–9	518 (32%)	15 (20%)		
10–18	550 (33%)	36 (48%)		
Initial symptoms			26.43	<.001
Fever	814 (50%)	31 (41%)		
Cough	548 (33%)	17 (23%)		
Fever + cough	224 (14%)	10 (13%)		
Other	51 (3%)	17 (23%)		
WBC (*10^9^/L)	9.35 ± 3.45	9.58 ± 3.84	1.087	.246
HGB (g/L)	136.29 ± 10.40	139.60 ± 312.17	2.102	.036
PLT (*10^9^/L)	282.10 ± 76.78	288.53 ± 71.79	1.450	.147
LY (%)	29.32 ± 11.90	62.73 ± 12.78	8.184	<.001
NEU (%)	61.45 ± 12.98	27.71 ± 11.82	7.931	<.001
MONO (%)	7.17 ± 2.70	7.97 ± 3.77	1.016	.310
NLR	2.79 ± 2.24	2.53 ± 3.51	9.775	<.001
CRP (mg/L)	9.67 ± 12.46	15.76 ± 23.32	0.870	.384

CRP = C-reactive protein, HGB = hemoglobin, LY = lymphocyte percentage, MONO = monocyte percentage, MP = Mycoplasma pneumonia, NEU = neutrophil percentage, NLR = neutrophil-to-lymphocyte ratio, PLT = platelets, WBC = white blood cell counts.

### 3.2. Selection of the predictive factors by stepwise regression

**Figure 3.**
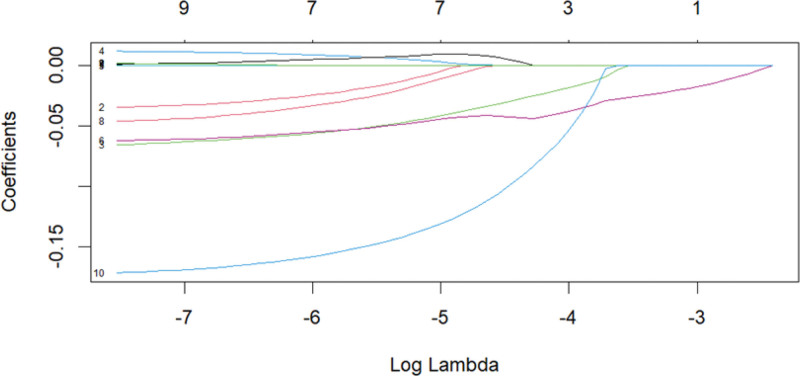
Variable selection by LASSO binary regression model. LASSO = Least Absolute Shrinkage and Selection Operator.

**Figure 4.**
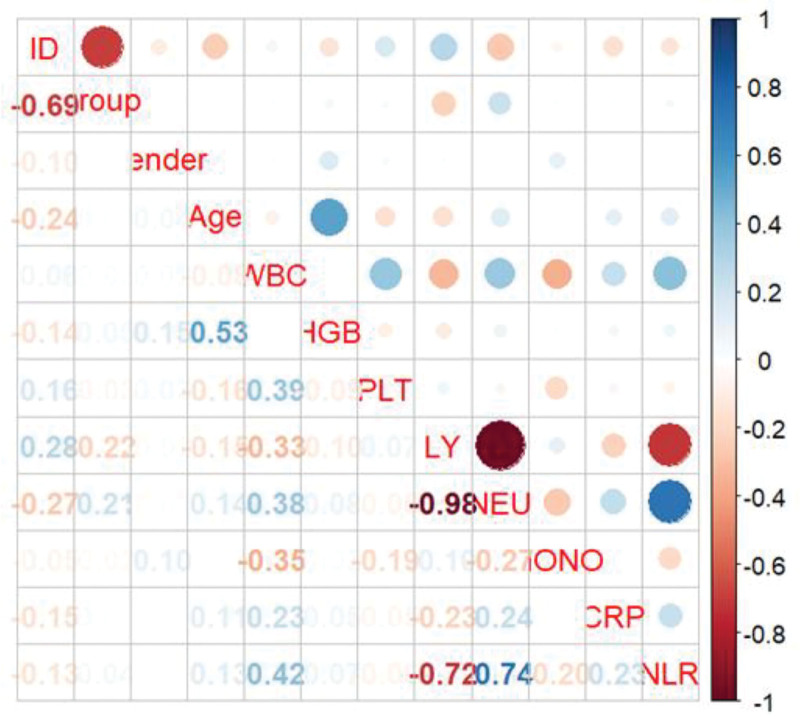
Spearman correlation analysis. WBC = white bloodcell counts, HGB = hemoglobin, PLT = platelets, LY = lymphocyte percentage, NEU = neutrophil percentage, MONO = monocyte percentage, NLR =neutrophil-to-lymphocyte ratio, CRP = C-reactive protein.

**Figure 5.**
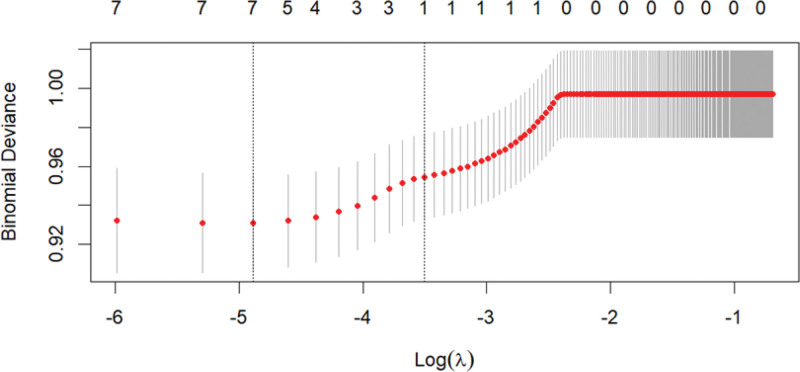
Selection of tuning parameter(λ) in the LASSO regression using 5-fold cross-validation via minimum criteria. LASSO = Least Absolute Shrinkage and Selection Operator.

**Figure 6.**
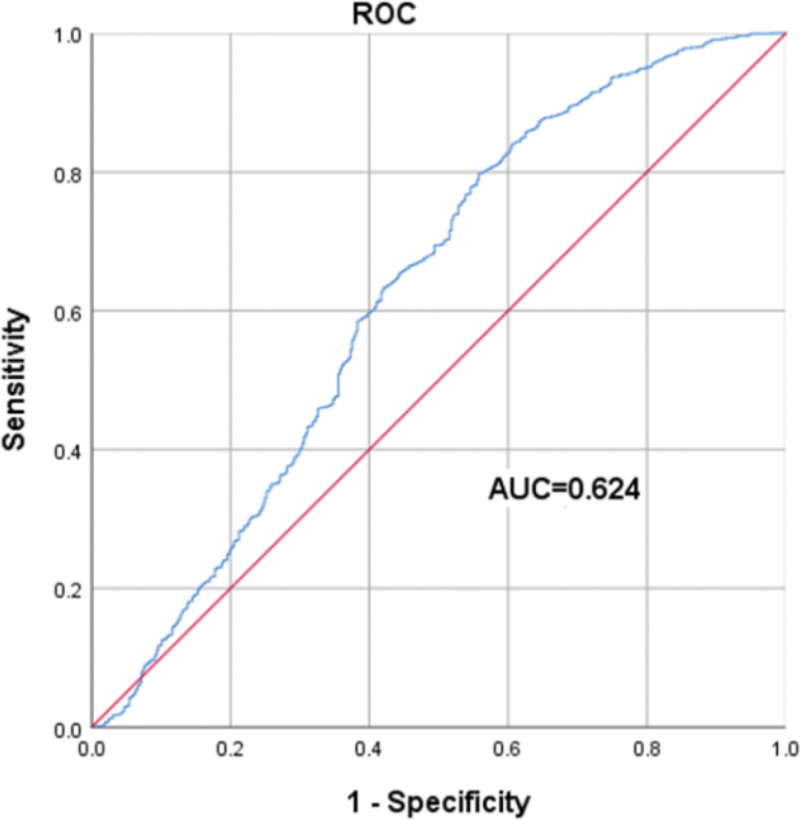
The ROC curve of the predictive values of NLR. A nomogram model to predict MPP was constructed based on the following 2 independent factors: WBC and NLR. AUC = area under the curve, NLR = neutrophil-to-lymphocyte ratio, ROC = receiver operating characteristic, WBC = white blood cell.

Taking the diagnosis of MP infection and non-MP infection as the dependent variables, factors with statistical significance in univariate analysis were selected for collinearity analysis in logistic regression. The Logistic step-by-step regression method showed that WBC and NLR might be valuable markers for predicting MP infection. Meanwhile, variables were screened using LASSO regression (Fig. 3). The Spearman correlation heatmap indicated that LY had collinearity with the occurrence of MP infection (Fig. 4), and both LY and NLR might be valuable markers for predicting MP infection (both of them *P* < .05, Table 2). Due to the collinearity between LY and MP infection, after excluding LY, one independent variable NLR was extracted (Fig. 5). The receiver operating characteristic (ROC) curve analysis showed that the area under the curve for the NLR in diagnosing MP infection was 0.624, with a cutoff of 1.36 (sensitivity was 0.798 and specificity was 0.558), as shown in Figure 6.

**Table 2:** Results of regression analysis.

Variable	Model 1 (Logistic step-by-step regression)			Model 2 (LASSO regression)		
	β	OR (95% CI)	*P*-value	β	OR (95% CI)	*P*-value
WBC	−0.069	0.933 (0.894–0.974)	<.001	-	-	-
LY	-	-	-	−0.063	0.939 (0.929–0.950)	<.001
NLR	−0.175	0.839 (0.784–0.894)	<.001	−0.201	0.818 (0.768–0.869)	<.001

LY = lymphocyte percentage, NLR = neutrophil-to-lymphocyte ratio, OR = odds ratio, WBC = white blood cell counts.

### 3.3. Establishment and validation of the nomogram model

**Figure 7.**
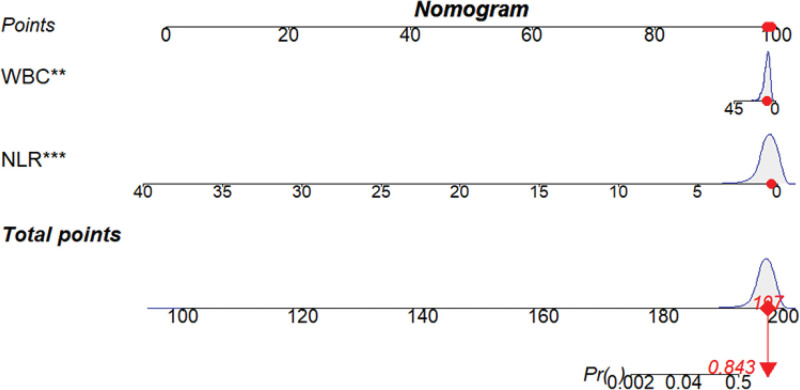
A nomogram model for predicting MPP. A nomogram model to predict MPP was constructed based on the following 2 independent factors: WBC and NLR. MPP = Mycoplasma pneumoniae pneumonia, NLR = neutrophil-to-lymphocyte ratio, WBC = white blood cell.

**Figure 8.**
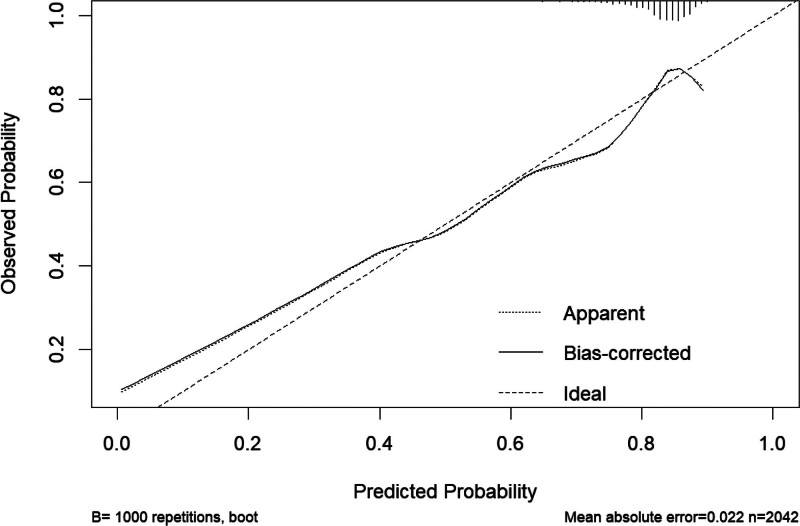
Calibration curve of the nomogram model. The observed apparent outcome (dotted line), the bias-corrected outcome (solid line), and the ideal outcome (dashed line) were presented.

Based on the screening results of Logistic step-by-step regression and LASSO regression, a Nomogram model for MP infection was constructed. As shown in Figure 7, a total of 2 independent variables were applied in this model, including WBC and NLR. The results indicated that the C-index of this model for predicting MP Infection was 0.654 (95% CI: 0.6227–0.687). As shown in Figure 8, the calibration curve indicates that the Nomogram Model performs well in predicting MP infection in children.

### 3.4. Comparison between RNA method and immunogold colloidal method in the diagnosis of MP infection

In the diagnosis of MP infection, the consistency between the ribonucleic acid (RNA) method and the immunogold colloidal method was poor (Kappa = 0.108, *P* < .05), as shown in Table 3.

**Table 3:** Consistency of 2 methods in detecting Mycoplasma pneumoniae.

		Immunogold colloidal method		
		Negative	Positive	
RNA	Negative	393	12	405
	Positive	1213	424	1637
Total		1606	436	2042

## 4. Discussion

MP is the smallest known prokaryotic microorganism capable of independent survival. It lacks a cell wall and is an important cause of community-acquired pneumonia in children.^[7]^ MP-related infections can occur throughout the year, with a higher incidence in autumn and winter in northern regions.^[8]^ The age of onset is mainly among preschool-aged and school-aged children.^[9]^

Although MP infections are usually mild, in recent years, due to the widespread use of macrolide antibiotics, the drug-resistance rate of MP has been increasing year by year, and the proportion of refractory cases is on the rise. There are several issues, such as the lack of specific and highly sensitive monitoring indicators in the early stage of the disease, rapid disease progression, complex and diverse imaging manifestations, and poor sensitivity of laboratory tests. These problems can easily lead to delays in diagnosis, and the disease may progress to severe pneumonia. Therefore, it is crucial to find easily monitored early-stage indicators.^[10]^

Our study included a total of 2042 children suspected of MP infection. And 1637 children were diagnosed with MP infection by the RNA method, and 405 children were non-Mycoplasma pneumonia-infected. The percentage of neutrophils in the non-Mycoplasma pneumonia-infected group was significantly lower than that in the MP-infected group; the differences between the 2 groups were significant (*P* < .001) in our study. In terms of the comparison of the percentage of LY and hemoglobin between the 2 groups of children, those in the MP-infected group were lower than those in the non-MP-infected group, and the differences were all statistically significant (*P* < .05) (Fig. 1).

Logistic step-by-step regression indicated that WBC and NLR might be valuable markers for predicting MP infection. Meanwhile, variables were screened using LASSO regression, and the Spearman correlation heatmap showed that there was collinearity between LY and the occurrence of MP infection (Fig. 2). The results showed that both LY and NLR might be valuable markers for predicting MP infection (both of them *P* < .05, Table 2). Therefore, LY needed to be excluded to eliminate the collinearity problem, and one independent variable, NLR, was extracted.

Some studies have shown that the levels of peripheral blood eosinophils and the NLR in patients with MP infection are higher than those in the non-infected group. The levels of blood eosinophils and NLR are closely related to MP infection in children, and both are of great value in diagnosing MP infection in children. Moreover, the combined diagnosis has a higher value.^[11]^ In addition, some research has demonstrated that high levels of C-reactive protein, D-dimer, the ratio of T-lymphocyte CD4+/ CD8+, age of onset, duration of fever, and LDH are early independent warning indicators for refractory MP pneumonia in children.^[12,13]^

The risk factors were screened based on Logistic stepwise regression and LASSO regression, including 2 independent variables: WBC and NLR, and a Nomogram model for predicting MP infection was established, as shown in Figure 7. Our research indicated that the C-index predicted by this model for MPP is 0.654 (95% CI: 0.6227–0.687). In addition, Figure 8 presents the calibration curve of the Nomogram Model for predicting MPP in children, which appears to be relatively ideal.

In clinical practice, NLR is easy to obtain, measure, and has relatively high economic benefits. It is well known that the important components of the immune system include neutrophils and LY. Neutrophils play a key role in the immune response of MP and are the first line of defense against infection.^[14]^ After MP infection, the neutrophil counts in peripheral blood,^[15]^ bronchoalveolar lavage fluid,^[16]^ and lung tissue^[17]^ increased significantly. Neutrophils interact with endothelial cells and platelets to build a bridge between inflammation and thrombosis.^[18]^ However, their excessive activation can lead to tissue damage.^[19]^ On the contrary, excessive inflammation can induce lymphocyte apoptosis and reduce lymphocyte counts.^[20]^

Furthermore, the research by Dan Li et al indicated that one of the important predictors of poor prognosis in patients with MPP is NLR, including the occurrence of adverse outcomes such as necrotizing pneumonia and refractory MPP. In the early stage of MPP, NLR can be used for simple and rapid prognosis prediction, and appropriate treatment can be initiated at an early stage. This study focused on the diagnosis of MP infection, and the early predictive indicators for the aggravation of the disease into refractory MP pneumonia have not been analyzed. In the future, the sample size can be further expanded, and follow-up efforts can be strengthened to analyze the risk factors for refractory MP pneumonia.

Our study also analyzed the consistency between the RNA method and the immunogold colloidal method for detecting MP infection. The results showed that the diagnostic consistency of the 2 methods was poor (Kappa = 0.108, *P* < .05). Few studies have compared the consistency of the 2 methods. A literature study with 270 children with MP infection pointed out that the positive rate of the immunogold colloidal method was only 23.33%, which is relatively low. In this study, the positive rate of the immunogold colloidal method was 26%, which is consistent with previous studies.^[21]^ The study by Huiwen L et al^[22]^ showed that the nucleic acid detection method from pharyngeal swabs has high sensitivity and application value in the etiological diagnosis of the acute phase of MP infection in children. The detection results of DNA fluorescence quantitative amplification and RNA isothermal amplification have a high degree of consistency and have more advantages compared with the detection of MP-IgM (enzyme-linked immunosorbent assay). In this study, the positive rate of the immunogold colloid method was significantly lower than that of the RNA method. The RNA isothermal amplification method has relatively higher sensitivity and anti-interference ability compared with the immunogold colloidal method. In addition, the 2023 Guidelines for the Diagnosis and Treatment of MP Pneumonia in Children included positive MP-DNA and MP-RNA test results in the diagnostic criteria for MP infection.

This study still has some limitations. First, this study used peripheral blood, and the determination result of HGB was unstable. Precision and accuracy tests were not carried out. Although the difference in HGB between the 2 groups was statistically significant, the absolute mean difference (136 vs 139 g/L) was small, suggesting limited clinical relevance. Therefore, this finding should be interpreted with caution and requires further verification. Second, there is a lack of imaging data for children with MP infection, and the risk factors for its further development into MP pneumonia are still unclear. Third, the study period was limited to 3 months (October to December 2023), which coincides with the peak season of MP infection in Northern China. Therefore, the seasonal distribution of cases may be biased, and the findings might not fully represent year-round epidemiological patterns. Fourth, as this was a retrospective study, potential selection bias and incomplete information could not be entirely avoided. Fifth, although we constructed a nomogram model for predicting MP infection, external validation was not performed, which limits the generalizability of the model. Future studies with prospective, multi-center, and year-round designs, incorporating imaging data and external validation of predictive models, are warranted to strengthen and extend these findings.

In conclusion, the consistency between the RNA method and the immunogold colloidal method in the diagnosis of MP infection is poor. Both the WBC and NLR are valuable markers for MP infection. In general, to improve the predictive value, multiple factors can be considered in combination in clinical practice. However, to better apply the above conclusions to clinical practice, large-scale, multi-center studies are still needed for verification.

## Author contributions

**Conceptualization:** Kun Zhang, Xiaodan Zhai, Ying Zhang, Xiaolin Xu, Xuejing Wang.

**Data curation:** Ying Zhang, Xiaolin Xu, Xuejing Wang.

**Formal analysis:** Kun Zhang.

**Supervision:** Xiaodan Zhai.

**Validation:** Kun Zhang, Xiaodan Zhai, Ying Zhang, Xiaolin Xu, Xuejing Wang.

**Visualization:** Kun Zhang, Xiaodan Zhai, Ying Zhang, Xiaolin Xu, Xuejing Wang.

**Investigation:** Kun Zhang, Xiaodan Zhai, Xiaolin Xu, Xuejing Wang.

**Methodology:** Kun Zhang, Xiaodan Zhai, Xiaolin Xu, Xuejing Wang.

**Funding acquisition:** Kun Zhang, Xiaodan Zhai, Xuejing Wang.

**Writing – original draft:** Kun Zhang, Xuejing Wang.

**Writing – review & editing:** Kun Zhang, Xuejing Wang.
